# Epigenetic Regulation of Thyroid Hormone Receptor Beta in Renal Cancer

**DOI:** 10.1371/journal.pone.0097624

**Published:** 2014-05-21

**Authors:** Anna Wojcicka, Agnieszka Piekielko–Witkowska, Hanna Kedzierska, Beata Rybicka, Piotr Poplawski, Joanna Boguslawska, Adam Master, Alicja Nauman

**Affiliations:** 1 Department of Biochemistry and Molecular Biology, Centre of Postgraduate Medical Education, Warsaw, Poland; 2 Genomic Medicine, Department of General, Transplant and Liver Surgery, Medical University of Warsaw, Warsaw, Poland; University of Navarra, Spain

## Abstract

Thyroid hormone receptor beta (*THRB*) gene is commonly deregulated in cancers and, as strengthened by animal models, postulated to play a tumor-suppressive role. Our previous studies revealed downregulation of *THRB* in clear cell renal cell carcinoma (ccRCC), but the culpable mechanisms have not been fully elucidated. Since epigenetic regulation is a common mechanism influencing the expression of tumor suppressors, we hypothesized that downregulation of *THRB* in renal cancer results from epigenetic aberrances, including CpG methylation and microRNA-dependent silencing. Our study revealed that ccRCC tumors exhibited a 56% decrease in *THRB* and a 37% increase in DNA methyltransferase 1 (DNMT1) expression when compared with paired non-neoplastic control samples. However, *THRB* CpG methylation analysis performed using BSP, SNaPshot and MSP-PCR consistently revealed no changes in methylation patterns between matched tumor and control samples. *In silico* analysis resulted in identification of four microRNAs (miR-155, miR-425, miR-592, and miR-599) as potentially targeting *THRB* transcript. Luciferase assay showed direct binding of miR-155 and miR-425 to 3′UTR of *THRB,* and subsequent in vivo analyses revealed that transfection of UOK171 cell line with synthetic miR-155 or miR-425 resulted in decreased expression of endogenous *TRHB* by 22% and 64%, respectively. Finally, real-time PCR analysis showed significant upregulation of miR-155 (354%) and miR-425 (162%) in ccRCC when compared with matched controls. Moreover, microRNA levels were negatively correlated with the amount of *THRB* transcript in tissue samples. We conclude that CpG methylation is not the major mechanism contributing to decreased *THRB* expression in ccRCC. In contrast, *THRB* is targeted by microRNAs miR-155 and miR-425, whose increased expression may be responsible for downregulation of *THRB* in ccRCC tumors.

## Introduction

Thyroid hormone receptors (TRs): TRα and TRβ, are ligand (3,5,3′-triiodothyronine, T3)-inducible transcription factors that mediate the cellular effects of thyroid hormones, namely its active form – T3. Since T3-dependent genes include numerous important regulators of cell cycle, such as mdm2, p53 and retinoblastoma [1],[2], actions of TRs contribute to maintenance of key cellular processes including proliferation, differentiation, apoptosis and metabolism [3]. TRs include 3 functional proteins: TRα1, TRβ1 and TRβ2, encoded by *THRA* and *THRB* genes. TRα1 and TRβ1 receptors are expressed in virtually all tissues, but their expression levels and functionality depend on the cell type and developmental stage of an organism. Disturbed activity of TRs, resulting from aberrances in expression or sequences of genes coding for TRs is a common phenomenon in human cancers. It leads to disturbed expression of T3-dependent genes and, in consequence, to severe impairment in cellular homeostasis. Those observations led to hypothesis on the tumor suppressive role of TRβ, further confirmed in several elegant studies performed in cell lines and mouse models [4],[5].

One of the cancers in which aberrances in TRβ function are frequently observed, is the most common type of renal tumors - clear cell renal cell carcinoma (ccRCC). Most interestingly, *THRB* gene resides within 3p21-25 chromosomal region, known as a hot spot for mutations in genes involved in ccRCC pathogenesis [6]. The culpable mechanisms identified so far include mutations, aberrant expression and deregulated splicing [7],[8],[9].

It has been shown that ccRCC is accompanied by epigenetic aberrances that may directly contribute to tumorigenic process, acting at early and precancerous phase of the multistage renal tumorigenesis [10]. Numerous recent studies indicate that epigenetic deregulation is among major causes of derailed actions of tumor suppressors in cancers, and most frequently identified mechanisms include DNA methylation and microRNAs. DNA methylation consists in addition of a methyl group to a cytosine preceding guanine (CpG) in DNA sequence, and is catalyzed by DNA methyltransferases (DNMTs): DNMT1, DNMT3A and DNMT3B. DNMT3A and DNMT3B are *de novo* methyltransferases, expressed prevalently during early stages of development [11] that sustain parental methylation patterns. DNMT1 is referred to as the maintenance methyltransferase, as it is responsible for maintaining methylation patterns during DNA replication [12]. Aberrant methylation in cancer is caused by impaired expression and function of DNMTs. Cancer cells usually exhibit overall genomic hypomethylation, leading to microsatellite instability and activation of genes involved in metastatic changes [13],[14]. Simultaneously, severe hypermethylation of regulatory regions of tumor supressors, DNA repair and cell cycle control genes leads to progression of cancer [15]. For ccRCC it was shown that numerous cases of downregulated expression of VHL tumor suppressor, inactivated in approx. 50% of ccRCC patients, result from hypermethylation of the gene’s promoter [16].

MicroRNAs (miRNAs) are short RNAs that inhibit expression of protein-coding genes by binding to complementary sequences in 3′UTRs (Untranslated regions) of their transcripts. The sequence responsible for this recognition encompasses nucleotides 2–8 of a miRNA and has to be fully complementary to the target sequence in mRNA [17]. Numerous cancers exhibit aberrant expression of microRNAs, leading to severe changes in the transcriptome and proteome of a cancer cell. For ccRCC, a number of microRNAs were shown to be deregulated in tumor [18]–[21]. Both DNA methylation and microRNA –dependent regulation have recently brought the attention as two mechanism with greatest potential for possible therapeutic interventions [22]. It was shown that tumor suppressor genes, silenced due to DNA hypermethylation, can be reactivated with demethylating agents. Furthermore, microRNA mimics and inhibitors are currently evaluated as therapeutic molecules that show promise in personalized anticancer medicine [23].

The role of epigenetic changes in *THRB* disturbances in cancer has been investigated in a few studies. The potential role of microRNA-dependent regulation in *THRB* silencing was suggested for ccRCC [9] and proven for papillary thyroid carcinoma [24]. Several other studies suggested DNA hypermethylation as a mechanism contributing to *THRB* silencing in leukemia and cancers of breast, lung, and thyroid [25]–[30].

Those studies as well as frequent epigenetic deregulations observed in ccRCC [31], prompted us to analyze the possible impact of DNA methylation and microRNA-dependent regulation on *THRB* expression in renal cancer.

## Materials and Methods

### Material

Tissue samples were obtained with the permission of the Bioethics Committee of the Centre of Postgraduate Medical Education in Warsaw from patients with clear cell renal cell carcinoma (ccRCC) (Table S1). Written informed consent was obtained from all patients. The samples were divided into two groups: cancer tissues (n = 35, T) and control tissues (paired normal tissue from the opposite pole of the malignant kidney (>5 cm from tumor) with no histological evidence of tumor; n = 35, C). ccRCC was diagnosed by histology according to WHO criteria [32].

The following cell lines were used in the study: 1) HK-2 (ATCC no.: CRL-2190), a proximal tubular cell line derived from normal kidney, immortalized by transduction with human papilloma virus 16 (HPV-16) E6/E7 genes. 2) UOK171 (UOB Tumor Cell Line Repository, provided by Dr. W Marston Linehan, NIH, NCI, Bethesda, USA, patent E-033-2010/0): derived from IV stage ccRCC tumor, spontaneously immortalized. 3) HeLa (ATTC nr CCL-cervix adenocarcinoma. All cell lines were cultured according to provider’s instructions.

### Real-time PCR

RNA was isolated and reverse transcribed as described previously [33]. 200 ng of RNA was used for reverse transcription. Real-time PCR was performed as described previously [33] using primers specific for transcripts of *THRB* (THRB-RT-F: GAACAGTCGTCGCCACATC and THRB-RT-R: GCTCGTCCTTGTCTAAGTAAC) and *DNMT1* (DNMT1-RT-F: TTATCCGAGGAGGGCTAC and DNMT1-RT-R: GGCTTCACTTCTTGCTTG). Reverse transcription and real-time PCR of microRNAs was performed as described previously [34] using Taq-man probes specific for hsa-miR-155 (Life Technologies, Carlsbad, CA, USA, cat. no. 002623) and hsa-miR-425 (Life Technologies, Carlsbad, CA, USA, cat. no. 001516). Relative quantification of each expressed miRNA was calculated using the standard 2−ΔCt method. Association between the microRNA and *THRB* expression in tissue samples was calculated using the R environment [35] and the multiple correlation analysis according to the formula:
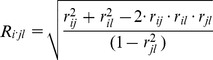



### 5-aza-2′deoxycytidine Treatment

UOK-171 cells were seeded onto 12-well plates (Corning, NY, USA) in amount of 5×10∧4 per well. The cells were cultured 24 hours in standard conditions, followed by addition of 100 mM or 10 mM 5-aza-2′deoxycytidine (5-aza-dC) (Sigma-Aldrich, Saint Louis, MO, USA). After 24 hours the cells were washed with PBS and used for RNA isolation.

### Analysis of *THRB* Methylation

For methylation analysis, we used the sequence of previously published *THRB* promoter [36], GenBank Acc. no. S37458.1), updated according to the sequence of chromosome 3 genomic contig (GenBank Acc. no. NT_022517.18). Due to disconcordance between the two deposited sequences, we cloned and directly sequenced DNA region encompassing *THRB* promoter.

To achieve this, genomic DNA was isolated from a non-tumorous kidney sample as described previously [37]. The region containing *THRB* promoter was amplified using primers THRB-HindIII-PromU (CGTAAAGCTTCATATGGGTAACACTGAGGGCATAGC) and THRB-HindIII-PromL (CGTAAAGCTTACTCCTTGGGCGAAGAGAGGC) and Hot Start Perpetual OptiTaq (EURx, Gdansk, Poland), under the following conditions: 95°C-10 min., 5 cycles: [96°C-35 s, 62°C-30 s, 73°C-80 s], 40 cycles: [96°C-40 s, 59°C-30 s, 72°C-80 s], final elongation: 72°C, 10 min. The obtained PCR product was cloned into pGEM-T vector (Promega, Madison, WI, USA) and sequenced. The obtained sequence was deposited in GenBank (Acc no. KF669869).

To identify CpG islands, we employed *in silico* analysis using CpG Plot [38] and CpG Island Searcher [39], CpG-rich region was detected in a sequence encompassing the promoter and 5′ UTR (exon 1) of TRβ transcript.

To analyze methylation of *THRB* CpG islands, 800 ng of genomic DNA was bisulphite-converted using Imprint DNA Modification Kit (Sigma-Aldrich, Saint Louis, MO, USA) according to manufecturer’s instructions. To predict the nucleotide changes resulting from bisulphite conversion, *THRB* sequence was *in silico* converted using Snake charmer (http://insilico.ehu.es/restriction/two_seq/snake_charmer.html) and used for design of primers.

To perform Bisulfite-Sequencing PCR (BSP), 100 ng of bisulphite-converted DNA was used, together with Perpetual Taq HOT START (EURx, Gdansk, Poland) polymerase and primers given in Table S2, under the following conditions: initial denaturation: 95°C, 10 min, 38 cycles: [denaturation: 95°C-15 s, annealing: 56°C or 57°C-15 s, elongation: 68°C-30 s], final elongation: 75°C, 15 min, final incubation: 4°C. The annealing temperature varied from 56°C to 67°C, depending on the used primers (Table S2). The obtained PCR products were directly sequenced by a commercial service (Genomed, Warsaw, Poland).

Methylation-specific PCR (MSP-PCR) was performed as described previously [26]. The sequences of primers are given in Table S3.

SNaPshot analysis was performed using SNaPshot Multiplex Kit (Applied Biosystems, Foster City, CA, USA) and primers given in Table S3 according to manufacturer’s instructions. The products were analyzed by a commercial service (Biote21, Krakow, Poland).

The specificity of primers used for methylation analysis was analyzed using MethPrimer [40].

### Analysis of microRNAs Targeting *THRB* Transcript

microRNAs potentially binding *THRB* 3′UTR were predicted using miRecords and DIANA–mirPath [41],[42] (Table S4). Next, PubMed was searched to find information on the expression of the predicted microRNAs in ccRCC. Only microRNAs with both reported increased expression in renal tumors and potentially targeting the *THRB* transcript were taken for further analysis.

To analyze the effect of microRNAs on indigenous *THRB* expression, UOK171 cells were seeded onto 12-well plates (Corning, NY, USA) using 5×10∧4 cells per well and transfected 24 hours later. Transfection mix was prepared as follows: 1) 2 µl Lipofectamine 2000 (Invitrogen/Life Technologies, Carlsbad, CA, USA) were mixed with 125 µl Opti-MEM (Gibco/Life Technologies, Carlsbad, CA, USA), and 2) 50 pmol microRNA mimics or inhibitors (Ambion/Life Technologies, Foster City, CA, USA, Table S5) were mixed with 125 µl Opti-MEM. After 10 minute incubation at room temperature the solutions were combined, incubated for additional 10 min, and added to the wells. After 6 hours transfection mixes were replaced with full medium. After 24 hours, the cells were harvested for RNA isolation.

For luciferase reporter assays, HeLa were chosen due to low endogenous expression of microRNAs (Figure S1). The cells were seeded onto 12-well plates (Corning, NY, USA) using 5×10∧4 cells per well and transfected 24 hours later. Transfection mix was prepared as follows: 1) 4 µl PEI 1µg/µl (Polyethylenimine, Polysciences, Warrington, PA, USA) were mixed with 125 µl Opti-Mem, and 2) 1 µg pGL3-luc-3′UTR-THRB plasmid (Jazdzewski et al., 2011) was mixed with 125 µl Opti-Mem. For reference plasmid, the following mixes were prepared: 1) 2 µl PEI with 62.5 µl Opti-Mem, and 2) 100 ng pRL-TK plasmid (Promega, Madison, WI, USA) with 62.5 µl Opti-Mem. After 20 minute incubation at room temperature the solutions were combined, incubated for additional 20 min and added to culture wells containing 700 µl medium without FBS. After 6 hours, the medium was replaced with transfection mixes containing microRNA mimics- prepared as described above. Information on microRNA mimics and inhibitors is given in Table S5. After 6 hours, transfection mixes were replaced with full medium; cells were lysed after 24 hours and analyzed in Dual Luciferase Reporter Assay (Promega, Madison, WI, USA) using Synergy 2 luminometer (BioTek, Winooski, VT, USA).

## Results

### The Expression of *THRB* and *DNMT1* is Changed in Renal Cancer

**Figure 1:**
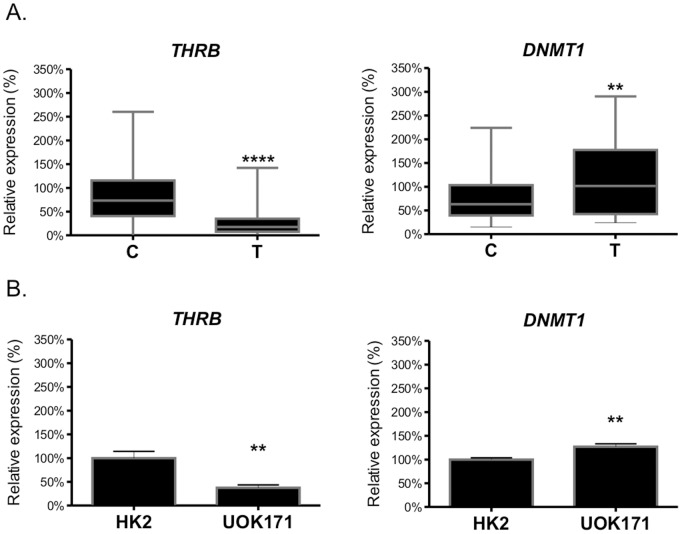
The expression of *THRB* and *DNMT1* in renal cancer. **A.** *THRB* and *DNMT1* mRNA expression in tissue samples: non-tumorous controls (C, n = 35) and paired ccRCC samples (T, n = 35). The data are shown as percent of control (C). Real-time PCR for each sample was performed in triplicates. Statistical analysis was performed using Wilcoxon matched pair test. **** p<0.0001. **B.** The expression of *THRB* and *DNMT1* mRNA in cell lines: HK2: proximal tubular cell line derived from normal kidney, UOK171: ccRCC cell line derived from IV stage tumor, spontaneously immortalized. The plots show results of five independent biological experiments, measured in triplicates. Statistical analysis was performed using t-test. ** p<0.01.

Real-time PCR analysis performed on ccRCC and paired non-tumorous control samples revealed significant changes in *THRB* mRNA expression (Figure 1). The expression of *THRB* was decreased by 56% (p<0.0001) in tumor when compared with control samples. This decrease of expression was found in 32 out of the 35 (91.4%) analyzed paired samples.

Real-time PCR analysis performed on cDNA from kidney cell lines confirmed decreased expression of *THRB* in renal cancer. *THRB* expression was 63% (p = 0.0036) lower in ccRCC UOK171 cell line when compared with non-cancerous HK2 cells (Figure 1).

Previous studies analyzing the expression of DNA methyltransferase 1 (DNMT1) in renal cancer brought conflicting results [43]–[45]. Therefore, we decided to analyze *DNMT1* expression in our study. DNMT1 mRNA levels were increased in 22 out of 35 analyzed ccRCC samples when compared with paired controls (Figure 1). The mean DNMT1 expression in tumor tissues was increased by 38% when compared with paired control tissue samples (p = 0.0098). In cell lines, DNMT1 expression was increased by 27% in UOK171 when compared with non-tumorous HK2 cell line (p = 0.0059).

These results suggested that increased *DNMT1* levels in ccRCC samples could possibly result in elevated CpG methylation leading to downregulation of *THRB* expression.

### Analysis of *THRB* CpG Methylation in Renal Cancer

**Figure 2:**
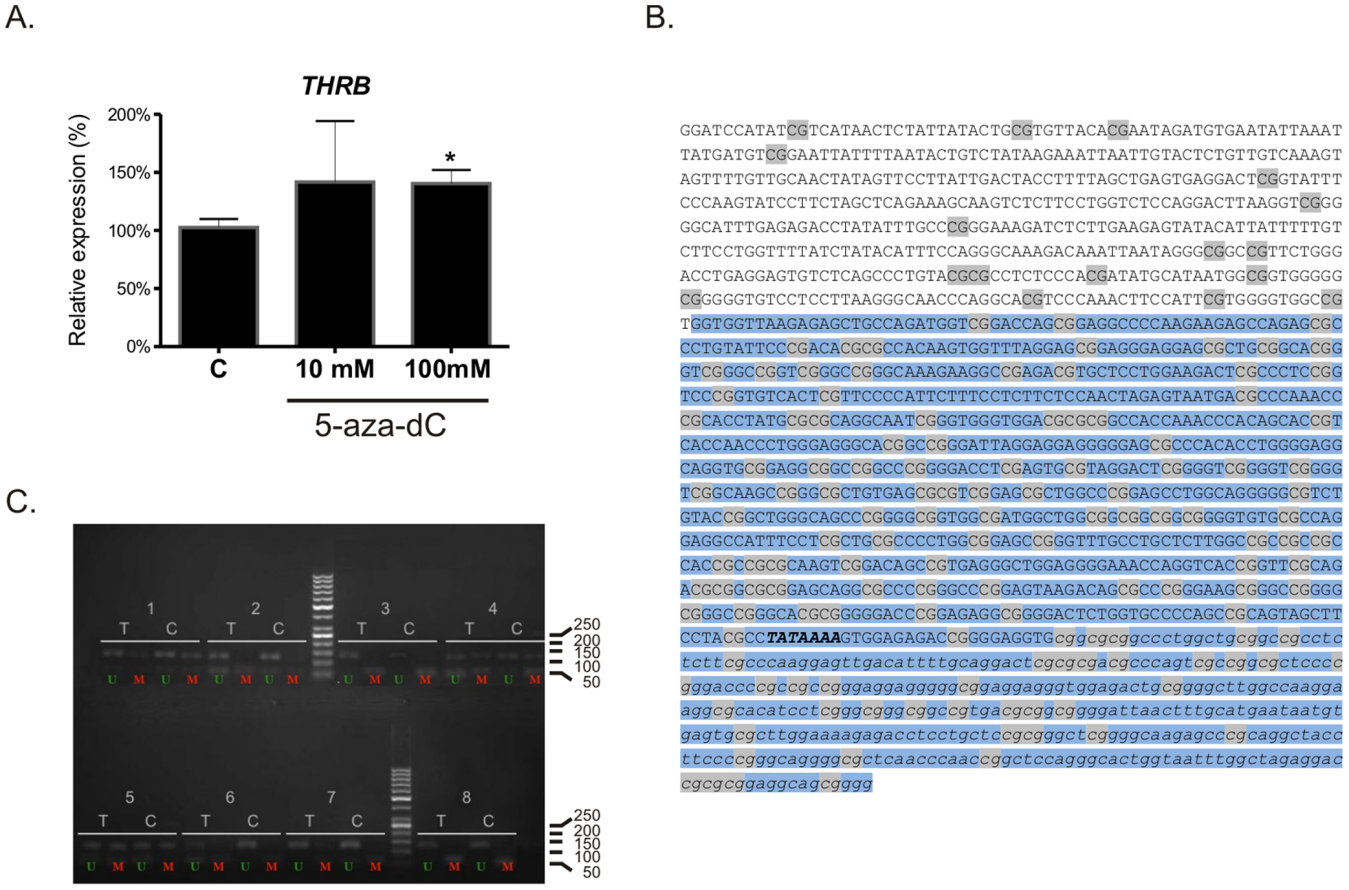
Analysis of *THRB* CpG methylation. **A.** The effect of 5-aza-2′ deoxycytidine (5-aza-dC) on *THRB* mRNA expression in UOK171 cell line. The results are shown as percent of control (the cells cultured without 5-aza-dC supplementation). The plot shows results of three independent biological experiments, measured in triplicates. Statistical analysis was performed using t-test. *p<0.05. **B.** The sequence of *THRB* gene with the promoter region (GenBank Acc.no. KF669869). CpG island, encompassing the promoter and 1^st^ exon (region −873 to +355) is shadowed blue. TATA box is bolded. CpG dinucleotides are shadowed gray. Lower case letters indicate 1^st^ exon of the TRβ transcript. **C.** Representative electrophoretic analysis of MSP-PCR. U: PCR products obtained with primers specific to unmethylated sequence; M: PCR products obtained with primers specific to methylated sequence. C: control samples, T: tumor samples.

To check whether the decreased *THRB* expression could result from CpG hypermethylation, UOK171 cells were cultured in the presence or absence of DNMT1 inhibitor, 5-aza-dC. Treatment with 5-aza-dC resulted in a statistically significant (p = 0.0124), 37% increase of *THRB* expression when compared with control, untreated cells (Figure 2A).

To determine whether restoration of *THRB* expression in renal cancer is directly regulated by promoter methylation, *THRB* sequences were analyzed using CpG Plot and CpG Island Searcher. Both programs predicted a CpG-rich region within the promoter and 5′UTR of *THRB* gene, encompassing nucleotides −873 to +355 (Figure 2B). Next, the predicted region was analyzed using bisulphite sequencing PCR (BSP). To this end, DNA samples from 15 pairs of ccRCC tissues and corresponding non-tumorous control kidney samples were bisulphite-converted and directly sequenced. Efficient bisulphite conversion was confirmed in PCR reactions with the use of primers designed to target native *THRB* promoter and not amplify bisulphite-converted DNA. Control amplification reactions performed on bisulphite converted DNA produced no amplification product (control primer sequences are given in Table S2). This analysis revealed lack of differences in methylation patterns between paired control and tumor samples from the same patient (Figure S2). To verify the results of BSP, the bisulphite converted DNA was additionally analyzed using SNaPshot and MSP-PCR, a highly sensitive method allowing for detection of 0.1% methylated alleles of a given CpG island locus [46] (Figure 2C). These analyses confirmed that there were no changes in CpG methylation patterns in ccRCC samples when compared with paired control samples. Interestingly, MSP-PCR results suggested that methylation patterns varied between different patients, being rather patient-specific than influenced by the disease status.

In conclusion, these results suggested that although changes in DNMT1 activity may contribute to alterations of *THRB* expression in cell cultures, the disturbed expression of *THRB* in ccRCC tissue samples is rather not a result of aberrant, tumor-specific hypermethylation of the gene’s promoter.

### microRNAs miR-155 and miR-425 Directly Target *THRB* 3′UTR

To predict microRNAs potentially influencing *THRB* expression in renal cancer, two-step analysis was performed. Firstly, bioinformatic analysis using miRecords (a resource that combines 11 bioinformatic programs) and Diana-mirPath predicted nearly 600 microRNAs potentially targeting *THRB* 3′UTR. However, based on the assumption that silencing efficiency is the highest for microRNAs that lie within the closest proximity to either end of the 3′UTR [47] we specifically focused on microRNAs that complied with that rule. Subsequent search in PubMed allowed for selection of microRNAs that were reported as overexpressed in renal cancer [18]–[21]. Using the two-step approach, four microRNAs (miR-155, miR-425, miR-592, and miR-599) were selected for further analysis.

**Figure 3:**
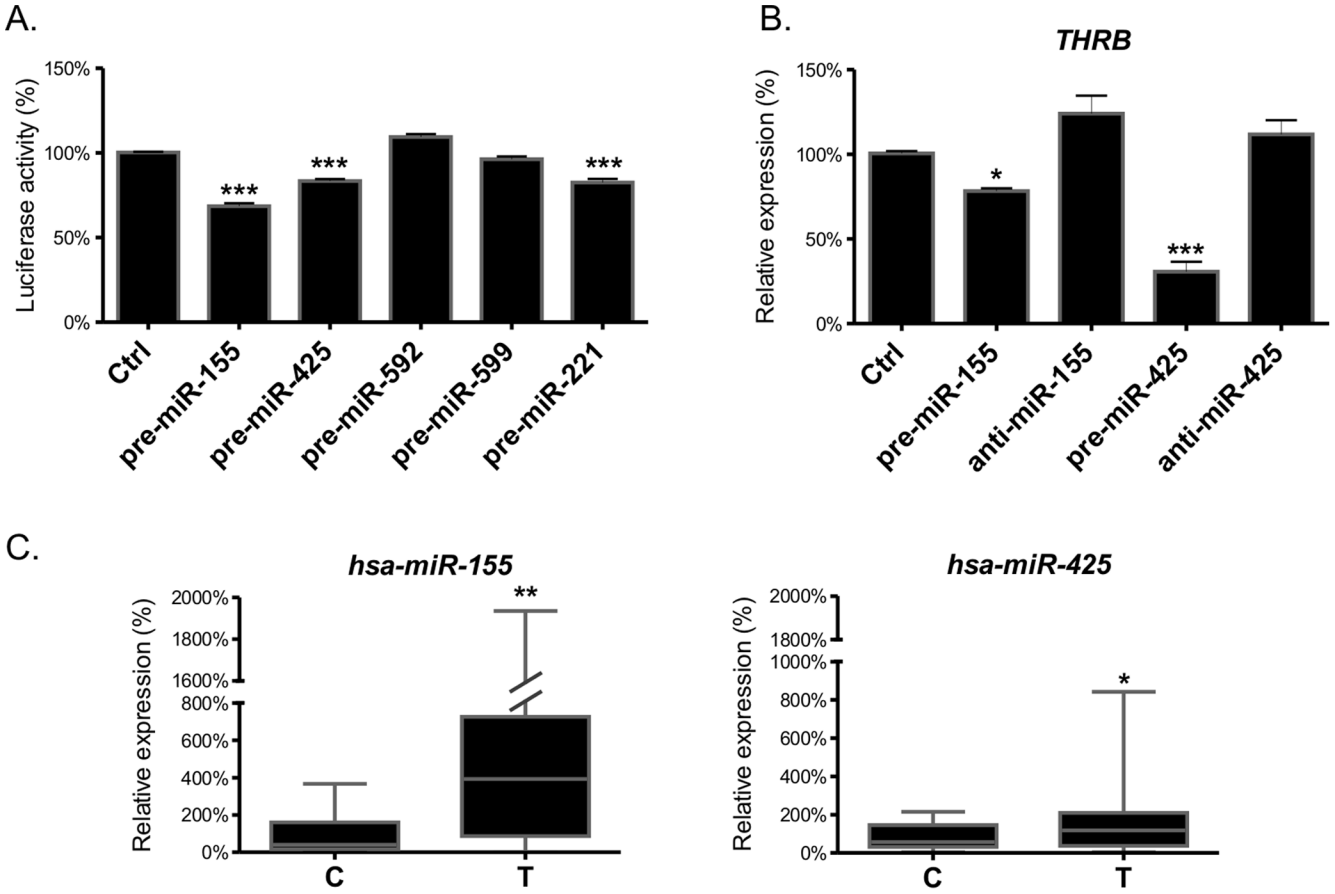
microRNAs targeting *THRB* transcript. **A.** The effect of microRNA mimics on the activity of luciferase expressed from a reporter plasmid pGL3-luc-3′UTR-THRB. HeLa cells were transfected using the reporter plasmid and respective microRNA mimics: pre-miR-155, pre-miR-425, pre-miR-599, or pre-miR-221. The results are shown as percentage of control (Ctrl, cells transfected with non-targeting pre-miR). The plot shows results of three independent biological experiments, measured in triplicates. The relative activity of Firefly luciferase was normalized to Renilla luciferase activity. Statistical analysis was performed using ANOVA followed by Dunnett’s multiple comparison test. *** p<0.001. **B.** The effect of miR-155 and miR-425 on endogenous *THRB* expression in renal cancer cells. UOK171 cells were transfected using respective microRNA mimics (pre-miRs) or inhibitors (anti-miRs), and *THRB* mRNA expression was analyzed using real-time PCR. The results are shown as percent of control (the cells transfected with non-targeting pre-miR). The plots shows results of three independent biological experiments measured in triplicates. Statistical analysis was performed using ANOVA followed by Dunnett’s multiple comparison test. *p<0.05, *** p<0.001. **C.** The expression of miR-155 and miR-425 in nontumorous controls (C, n = 35) and paired ccRCC tissue samples (T, n = 35). The results are shown as percent of control. Real-time PCR for each sample was performed in triplicates. Statistical analysis was performed using Wilcoxon matched pair test. *p<0.05, **p<0.01.

In order to find whether the chosen microRNAs indeed bind the 3′UTR of *THRB*, HeLa cells were transfected using synthetic microRNAs and a reporter construct pGL3-luc-3′UTR-*THRB* (Figure 3). HeLa cells were chosen for this analysis due to low endogenous expression of *THRB* and analyzed microRNAs. Transfection of cells with pre-miR-155 or pre-miR-425 resulted in 32% or 17% decrease of luciferase activity, respectively, when compared with control miRNA mimics (p<0.001). No changes in luciferase activity were observed in cells transfected with pre-miR-592 or pre-miR-599. Pre-miR-221, previously reported to target *THRB* 3′UTR [25] was used as positive control and confirmed the efficiency of miRNA-mediated silencing (Figure 3).

### miR-155 and miR-452 Influence *THRB* Expression in Renal Cancer Cells

To subsequently analyze the effect of miR-155 and miR-425 on endogenous *THRB* expression, renal cancer-derived UOK171 cell line was transfected using either microRNA mimics (pre-miR-155 or pre-miR-425) or microRNA inhibitors (anti-miR-155 or anti-miR-425) (Figure 3). Transfection resulted in a statistically significant decrease in *THRB* expression by 22% for pre-miR-155, p<0.05, and by 64% for pre-miR-425, p<0.001, when compared with control microRNA (Figure 3). Transfection of cell lines with microRNA inhibitors did not result in a statistically significant change of *THRB* expression, although a trend for increased expression was seen (Figure 3). This observation can result from the fact that levels of miR-155 and miR-425 are highly elevated in UOK171 cell line, thus inspite of numerous concentrations tested, the amount of microRNA inhibitors could be insufficient for efficient blocking of microRNA activity.

These studies suggested that miR-155 and miR-425 directly influence *THRB* expression in renal cancer cells.

### The Expression of miR-155 and miR-425 is Elevated in Renal Cancer and Correlates with Decreased Expression of *THRB*

In order to check whether tumor-specific changes of miR-155 and miR-425 could affect *THRB* expression in ccRCC tumors, the expression of both microRNAs was analyzed in 25 ccRCC tissue samples and 25 corresponding non-tumorous kidney samples (Figure 3). The expression of both microRNAs was statistically significantly increased (by 354%, p = 0.0072 for miR-155 and by 62%, p = 0.041 for miR-425). Simultaneously, expression of both miRs was negatively correlated with expression of *THRB*. Although the correlation coefficient was weak (R = 0.245, p = 0.0075) in control samples, the correlation was significantly stronger in tumors (R = 0.469, p = 0.0225). Concomitantly, there was also a significant correlation between the T/N ratio of miRNA and *THRB* expression (R = 0.396, p = 0.002). These results indicate that *THRB* expression levels depend on the miR-155 and miR-425 expression and deregulation of miRs is linked with lowered *THRB* levels in ccRCC (Figure S3).

## Discussion

In this study, using three different methods, we showed that in the analyzed samples of renal cancer aberrant *THRB* expression does not result from tumor-specific changes in DNA methylation of *THRB* promoter region. Rather, the expression of *THRB* in ccRCC is affected by microRNAs, miR-155 and miR-425 that directly bind to *THRB* 3′UTR, as suggested by the observation that elevated expression of these microRNAs in ccRCC is accompanied by downregulation of *THRB*.

Hypermethylation of *THRB* promoter was reported in several neoplasms, including breast, thyroid and lung cancers and leukemia [25]–[30]. Interestingly, the studies showed that methylation rates of *THRB* were highly variable among patients, since hypermethylated *THRB* was detected in 25–80% of analyzed tumors. Furthermore, only half of the studies on *THRB* methylation was performed on both tumor and paired non-tumorous control tissues. Iwasaki et al. examined 116 non-small cell lung cancers, including 6 samples for which paired adjacent control tissues were analyzed. Out of the 116 tumor samples, 54 revealed hypermethylation of *THRB*. When 6 paired tumor versus control samples were compared, *THRB* was methylated in all tumors and unmethylated in all non-tumorous control samples. In two other studies analyzing breast cancer tumors and cell lines [25],[29] hypermethylated *THRB* was detected in 80%-100% of tumors and c.a. 37% of paired samples. Moreover, the degree and pattern of methylation varied between samples derived from different patients. Similar interindividual variability was also observed in a study performed on thyroid tumors [30], in which paired normal tissue samples were not available and therefore not analyzed. Our study also revealed that *THRB* methylation significantly varied between tissue samples derived from different patients. These results suggest that lack of paired non-tumorous control samples may lead to a bias and result in classification of a patient-specific variations in *THRB* methylation as changes specific for cancer tissue.

Although *THRB* expression did not seem to be influenced by DNA methylation in our renal cancer samples, treatment of UOK171 cell line with 5-aza-2′ deoxycytidine resulted in increased expression of *THRB* gene. This suggests that *THRB* expression may be indirectly influenced by DNMT1 activity. According to previous studies, DNA methylation can indirectly affect transcription of a target gene in several mechanisms [48] including gene regulation by a reactivated transcription factor or signal transduction pathway that is affected by DNA methylation. Which of these mechanisms can affect *THRB* expression in renal cancer is currently unknown. Furthermore, we can not exclude the possibility that in other ccRCC patients and in the other ccRCC-derived cell lines, the *THRB* promoter methylation could influence *THRB* expression. This, hopefully, will be verified by future studies on ccRCC.

Another epigenetic mechanism that influences the expression of target genes is microRNA-dependent regulation of expression [49]. A single gene can be targeted by multiple microRNAs and single microRNA can regulate numerous target genes. In consequence, deregulation of miRNA expression that is associated with numerous cancers leads to a severe disruption of the cellular proteome and transcriptome. In this study we identified two microRNAs whose direct binding with *THRB* 3′UTR was confirmed in luciferase assay performed in HeLa cell line, used due to relatively low expression levels of both analyzed miRNAs. Analysis of expression in tissue samples revealed that upregulation of miR-155 and miR-425 in clear cell renal cell carcinoma is accompanied by downregulation of *THRB*. Insufficient specificity of the commercially offered anti-TRβ antibodies and extremely low expression levels of the TRβ protein did not allow for showing the miR-mediated effect on the TRβ protein level. It was however proven both in luciferase assay and *in* *vivo* study, in which the miRs downregulated indigenous *THRB* transcription.

A number of microRNAs regulating *THRB* expression were already described [9],[24],[50] and Jazdzewski et al. showed that microRNAs targeting *THRB* are strongly upregulated in papillary thyroid carcinoma, contributing to silencing of *THRB* in tumor. Importantly, the expression of microRNAs is highly specific for a tissue type, or characteristic for a specific disease state. Thus, microRNA signatures of ccRCC significantly differ from those of other cancers, including PTC, and this explains the fact why the obtained results were not overlapping. Since miR-155 and miR-425 analyzed in our study directly target 3′UTR of *THRB* and are overexpressed in ccRCC, it is possible that they contribute to decreased *THRB* expression in this cancer. The coefficient of multiple correlation, which is a measure of how well a given variable can be predicted using a linear function of a set of other variables, showed that *THRB* expression in tissue samples depends on the levels of both microRNAs. This regulation, together with previously reported altered splicing and mutations in *THRB* could directly lead to deregulation of thyroid receptor beta in renal cancer.

Interestingly, it was recently shown that DNA methylation and microRNA-dependent regulation are the mechanisms that complementarily regulate gene expression [51]. The study revealed a negative correlation between the level of promoter region methylation and a number of miRNA target sites in 3′UTR of a gene. Apparently, *THRB* belongs to the group of genes with long 3′UTRs, indeed targeted by numerous microRNAs [9],[24],[50]. This supports the notion on the important role of microRNA-dependent regulation of *THRB*, and on the possible role of deregulation of this mechanism in neoplastic process.

Both microRNAs analyzed in our study have well established role in tumorigenesis. For instance, aberrant expression of miR-425 was reported in hyperdiploid multiple myeloma, glioblastoma, breast, thyroid and prostate cancers [52]–[56]. Those aberrations in miR-425 expression can directly influence tumorigenesis, e.g. by affecting the expression of oncogenes [52]. Finally, in accordance with our study, upregulated expression of miR-425 was reported in renal cancer [57] and was proposed as one of markers differentiating between normal kidney and renal tumors [20].

MiR-155 is a well known oncogene which is overexpressed in a broad panel of neoplastic lesions, including cancers of breast, colon, cervix, pancreas, lung, and kidney, as well as in lymphomas and leukemias [19],[58],[59]. Most interestingly, it was recently shown that miR-155 targets VHL tumor suppressor and promotes angiogenesis in breast cancer [60]. VHL plays a pivotal role in ccRCC tumorigenesis and is mutated or silenced in more than half of sporadic clear cell renal cell carcinomas [61]. Intriguingly, VHL itself regulates the expression of miR-155 in ccRCC [62], suggesting a tumor suppressor-oncogene feedback regulation between VHL and miR-155. Moreover, the role of miR-155 in silencing of a tumor suppressor, revealed in this study, additionally supports the notion on the oncogenic role of this miRNA.

In conclusion, we identified two novel microRNAs, miR-155 and miR-425, that target *THRB* transcript and downregulate its expression in renal cancer. In contrast, CpG methylation is rather not the main mechanism directly contributing to deregulated *THRB* expression in renal cancer. The possible indirect effects of changes in DNMT1 activity on *THRB* expression need further evaluation.

## Supporting Information

Figure S1 **Analysis of expression of microRNAs miR-155 and miR-425 in HeLa and UOK171 cells.** The results show analysis of expression from cells cultured in three 25 cm^2^ bottles, normalized to U6 snRNA. Real-time PCR for each sample was performed in triplicates. Statistical analysis was performed using unpaired t test. **p<0.01, *p<0.001.
(TIF)

Figure S2 **Analysis of** ***THRB*** **CpG methylation.** Representative result of BSP. The chromatograms show results of sequencing performed on tumor (upper panel) and control (lower panel) samples taken from the same patient.
(PDF)

Figure S3 **Correlation between the ratio of miR-155 and miR-425 and** ***THRB*** **expression in tumor vs control samples.** Multiple correlation plots showing the association between the changes of microRNA and THRB levels in ccRCC samples. Lowered THRB expression in ccRCC when compared to paired control tissue is correlated with increased levels of miR-155 and miR-425.
(TIF)

Table S1 **Patient characteristics.** Information on 35 ccRCC patients included in the study: histopathological diagnosis, age at disease onset and gender.
(DOCX)

Table S2 **Primers used for BSP of** ***THRB*** **promoter.**
(DOCX)

Table S3 **Primers used in MSP-PCR and SNaPshot.**
(DOCX)

Table S4 **microRNAs predicted to bind 3′UTR of** ***THRB*** **based on miRecords database.**
(XLS)

Table S5 **microRNA mimics and inhibitors used in the study.**
(DOCX)
