# Supplementary figures and images for: Epigenetic Regulation of Thyroid Hormone Receptor Beta in Renal Cancer
Source: PLoS One. 2014 May 21;9(5):e97624. doi: 10.1371/journal.pone.0097624 (PMC4029725; doi:10.1371/journal.pone.0097624)

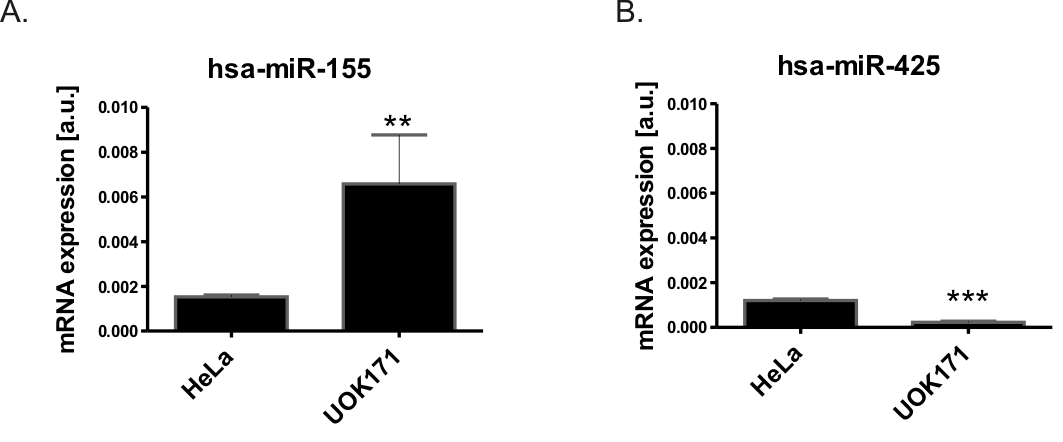

Supplement: Figure S1 — Analysis of expression of microRNAs miR-155 and miR-425 in HeLa and UOK171 cells. The results show analysis of expression from cells cultured in three 25 cm2 bottles, normalized to U6 snRNA. Real-time PCR for each sample was performed in triplicates. Statistical analysis was performed using unpaired t test. **p<0.01, *p<0.001. (TIF) [file pone.0097624.s001.tif]

1T

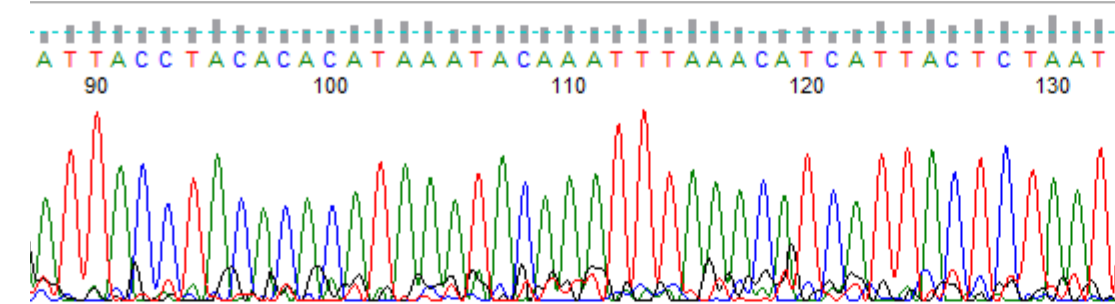

1N

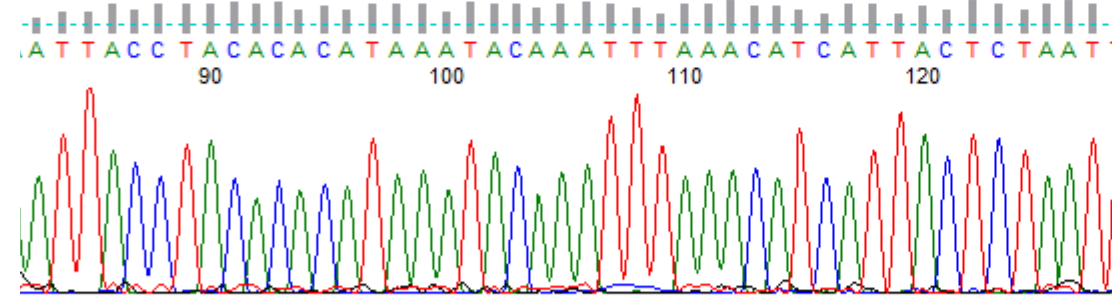

2T

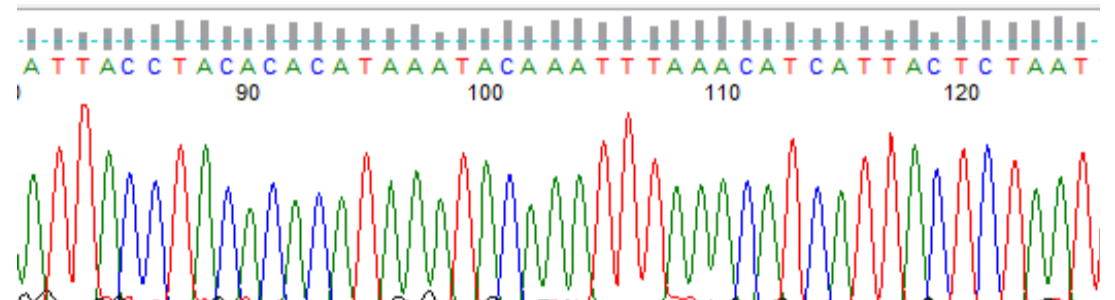

2N

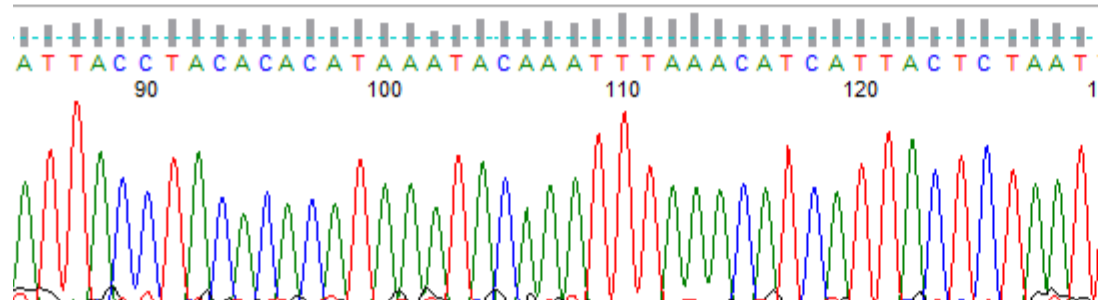

3T

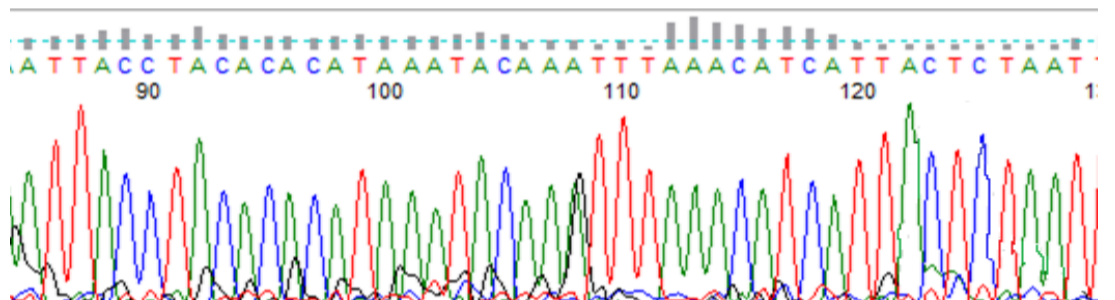

3N

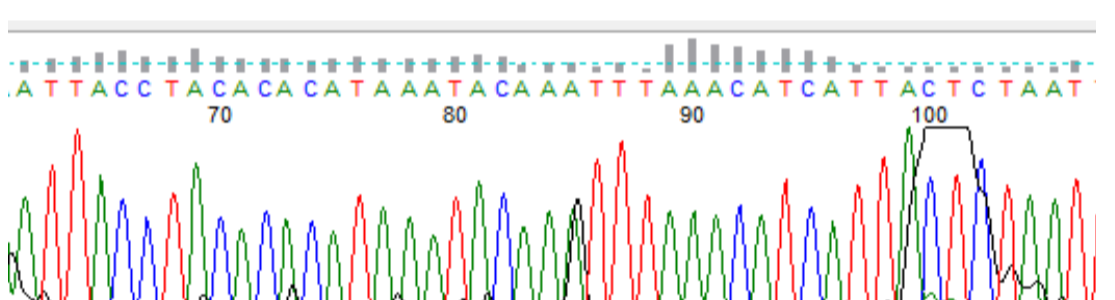

4T

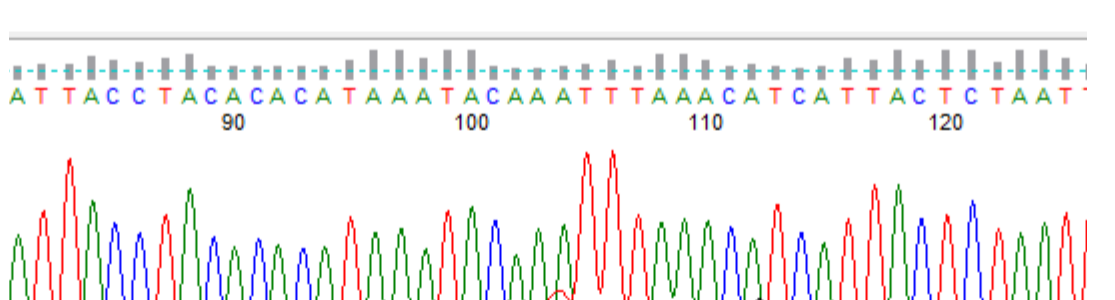

4N

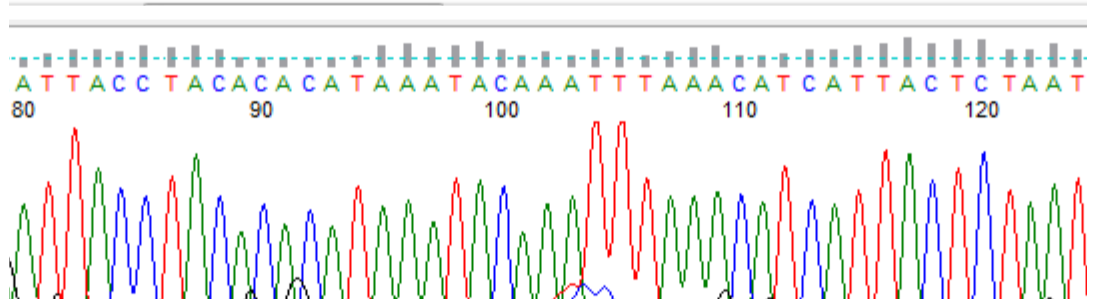

5T

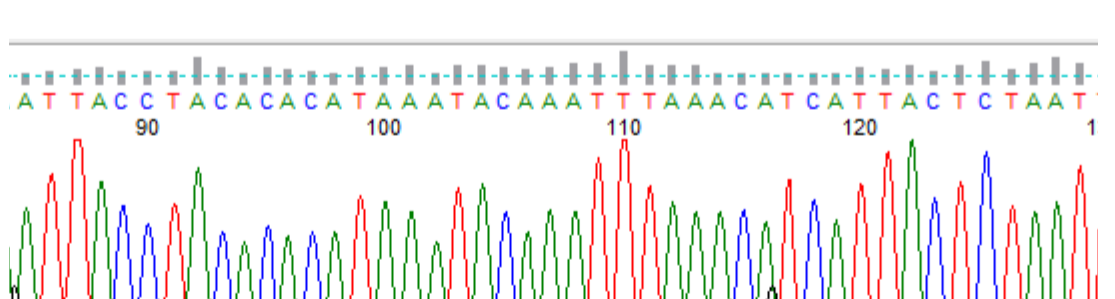

5N

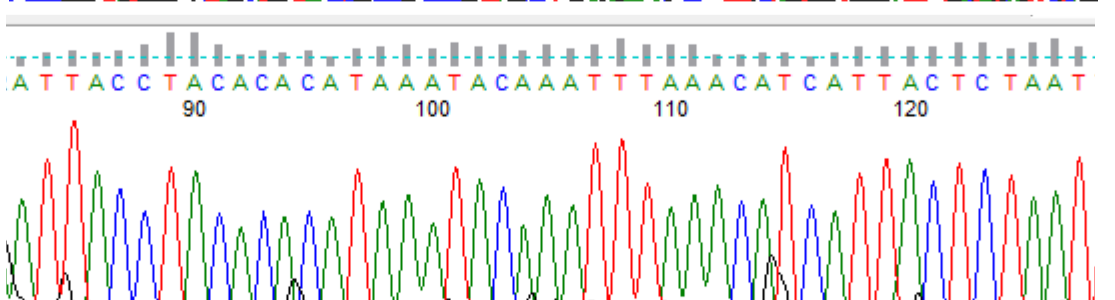

6T

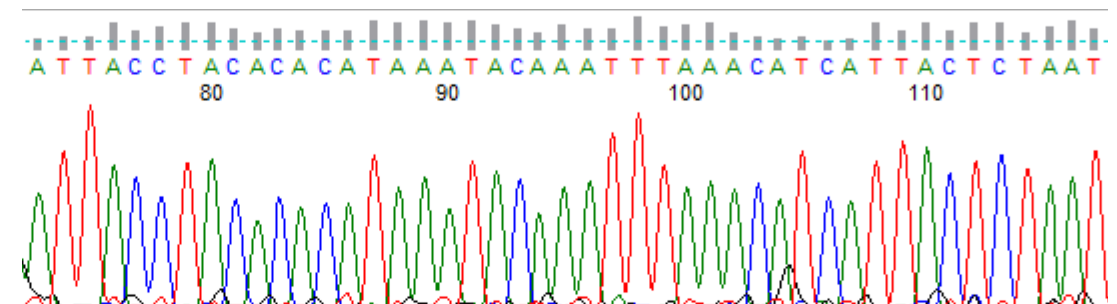

6N

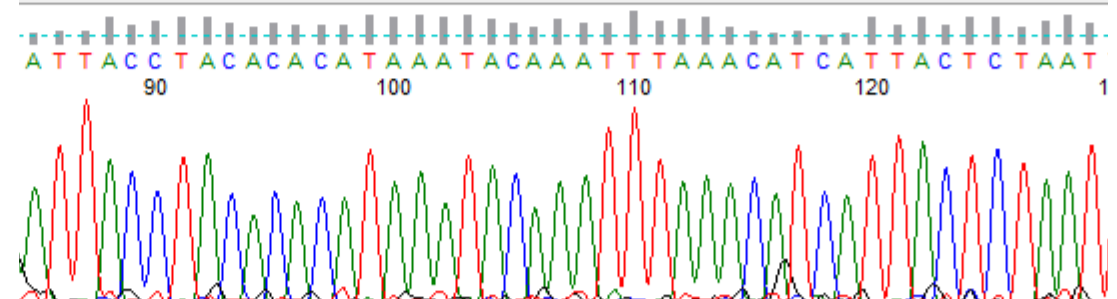

7T

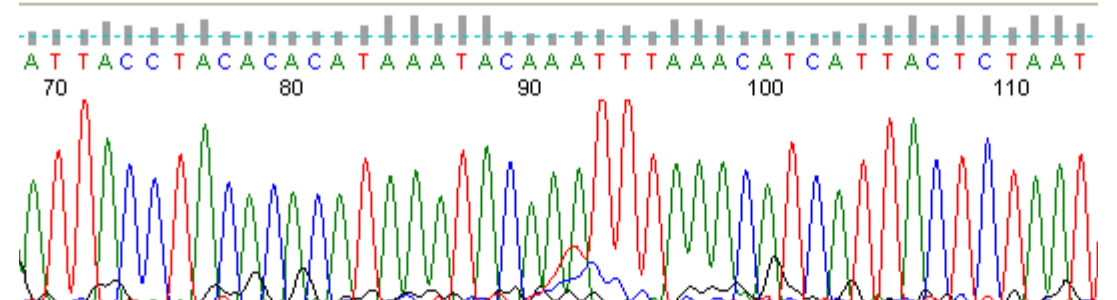

7N

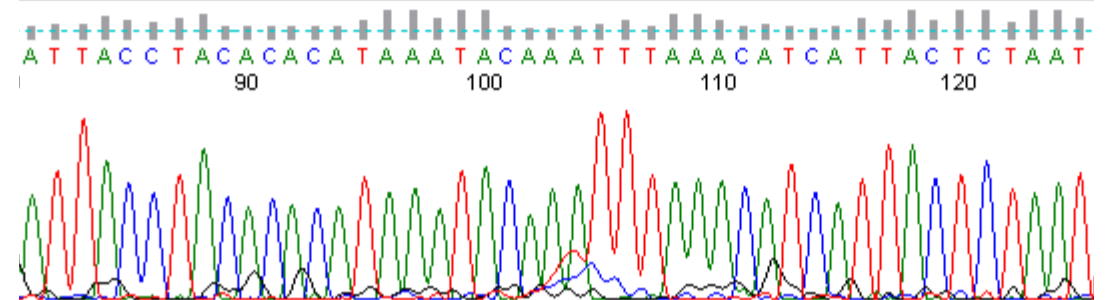

8T

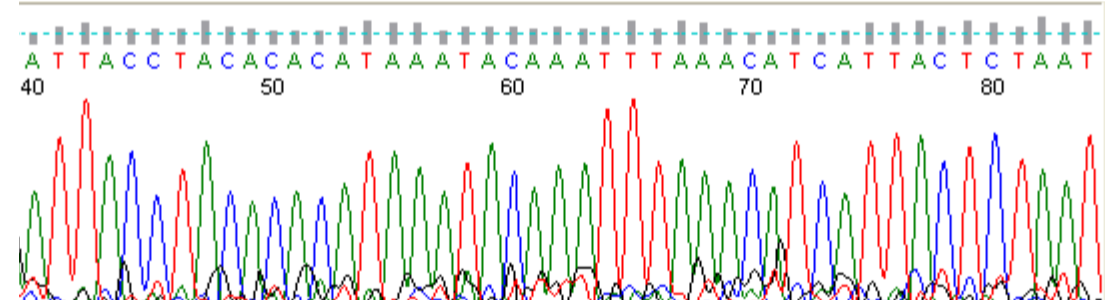

8N

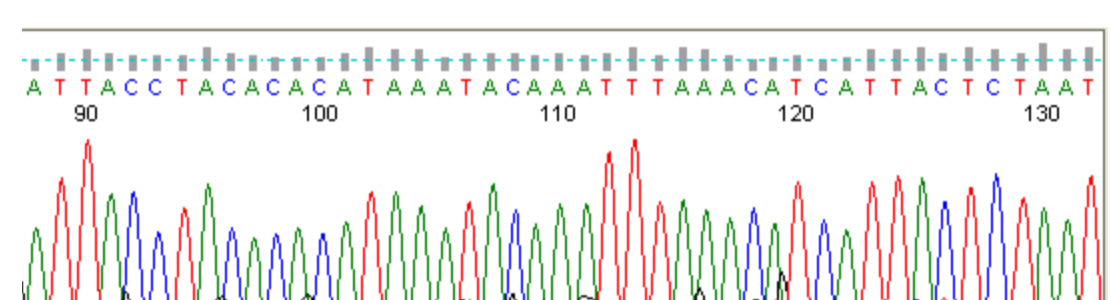

9T

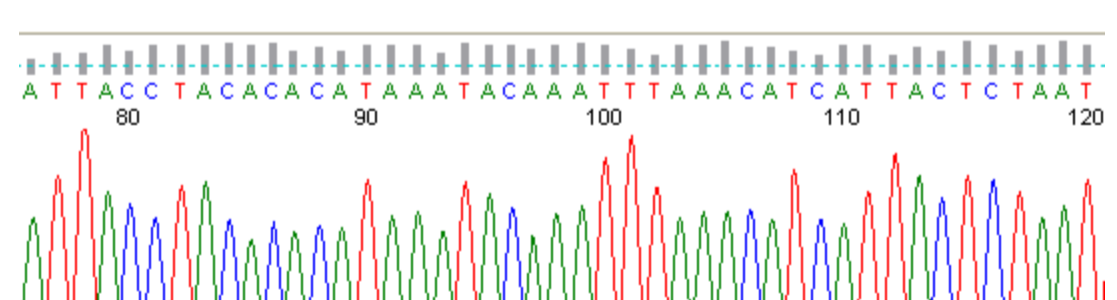

9N

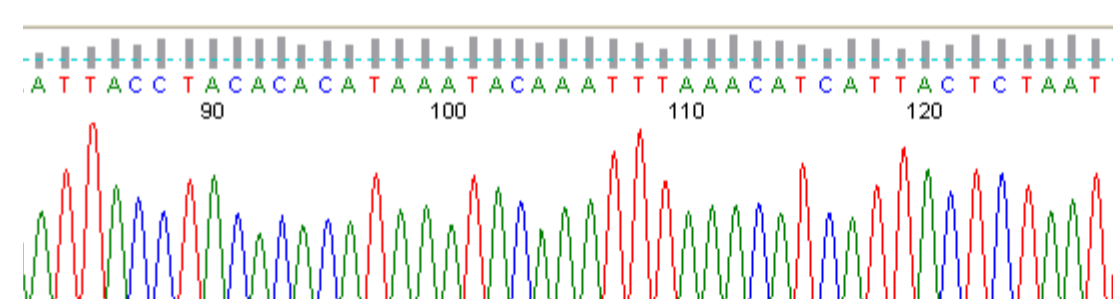

10T

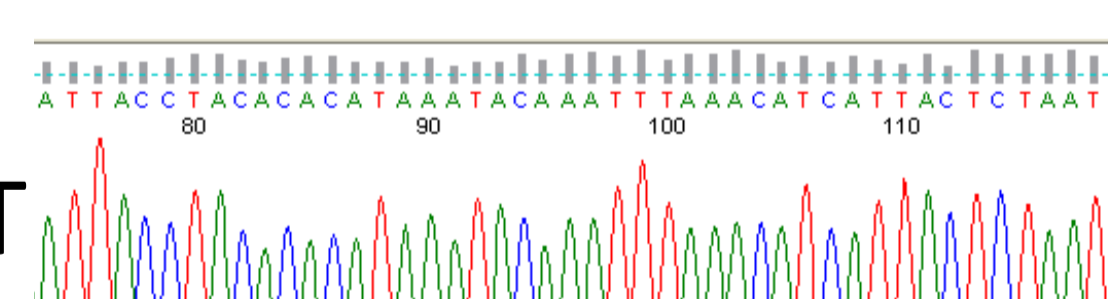

10N

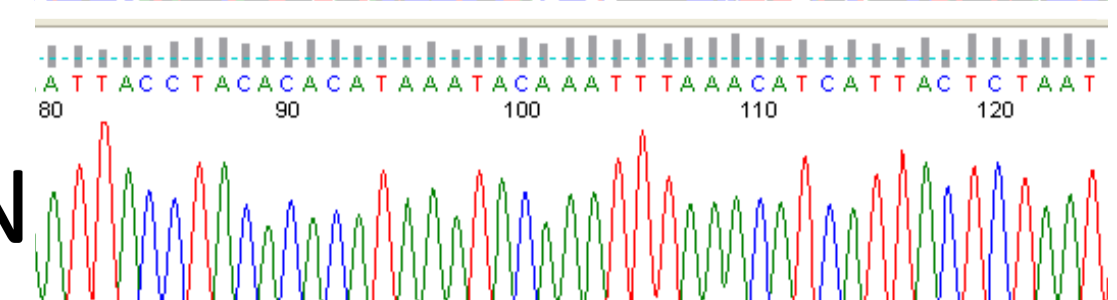

11T

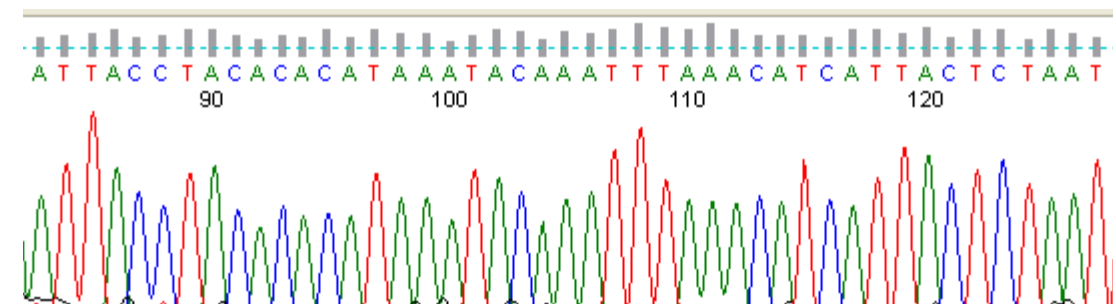

11N

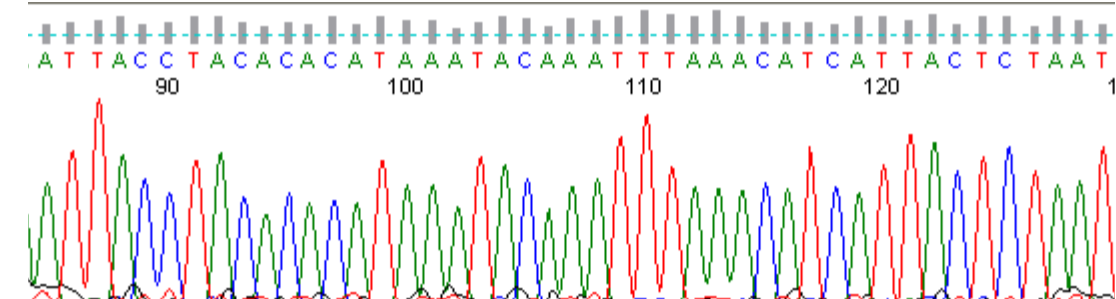

12T

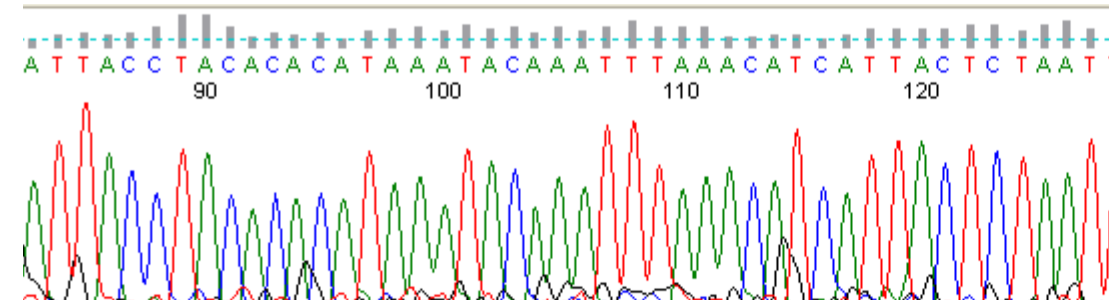

12N

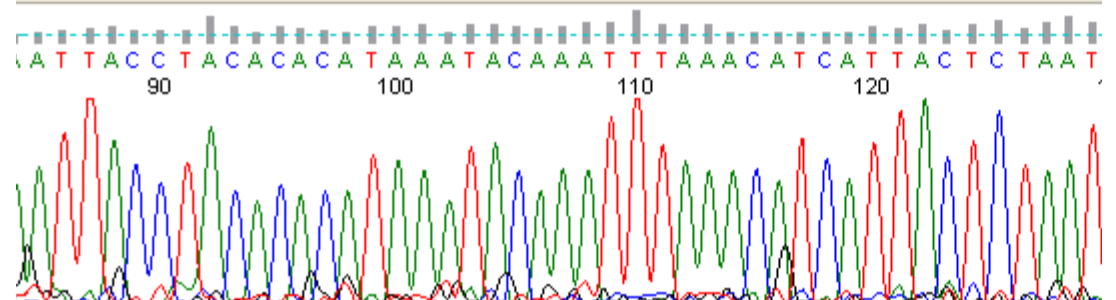

13T

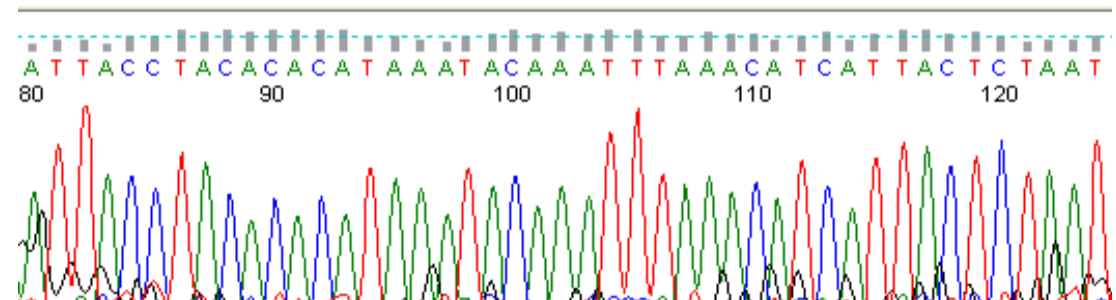

13N

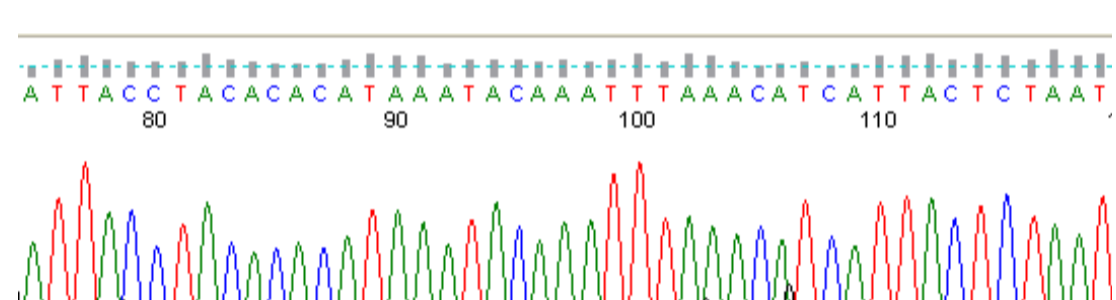

14T

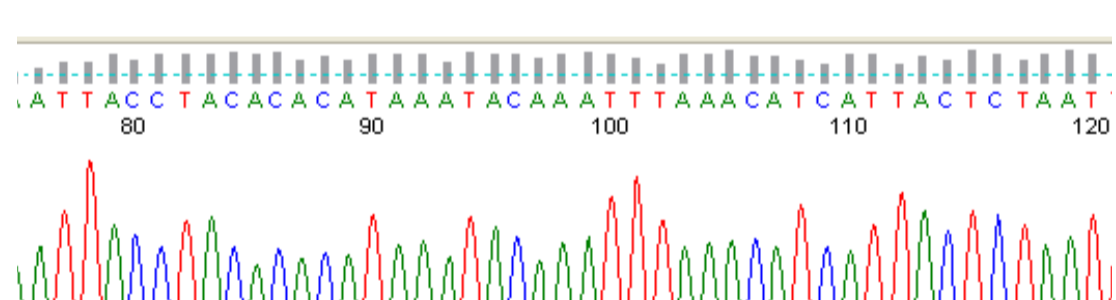

14N

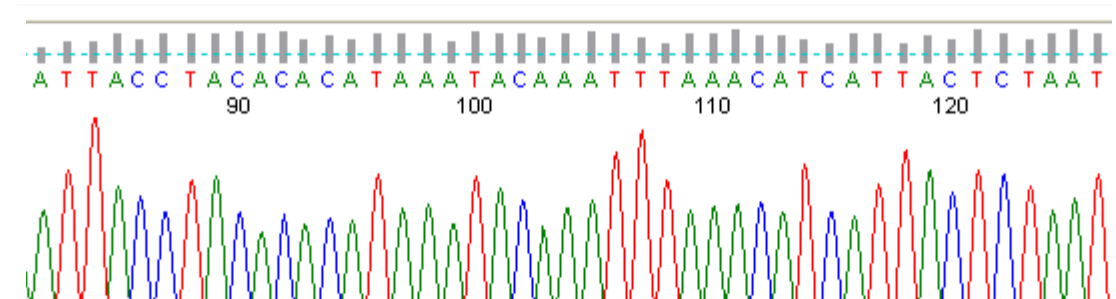

15T

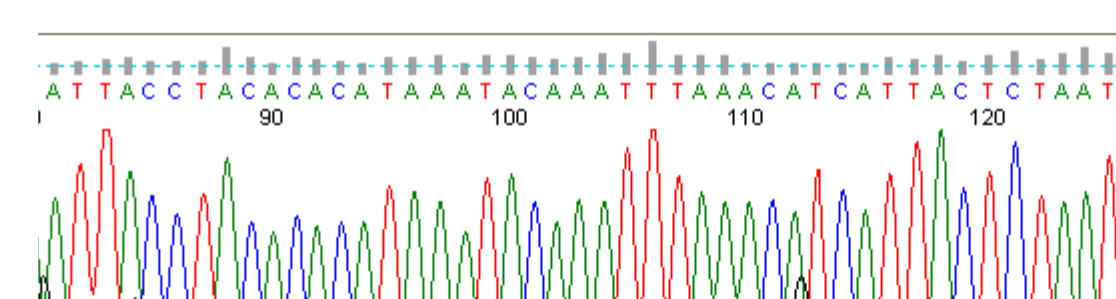

15N

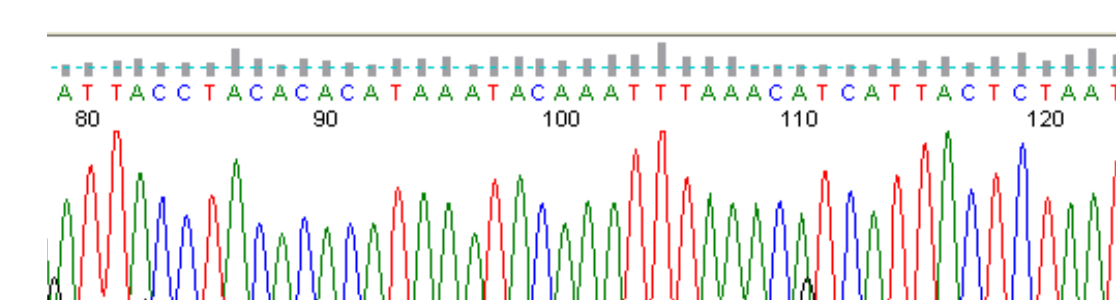

Supplement: Figure S2 — Analysis of THRB CpG methylation. Representative result of BSP. The chromatograms show results of sequencing performed on tumor (upper panel) and control (lower panel) samples taken from the same patient. (PDF) [file pone.0097624.s002.pdf]

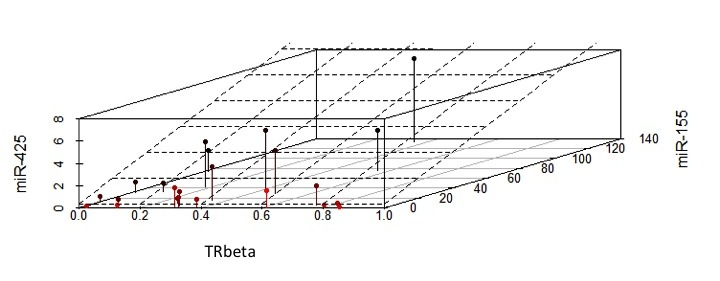

Supplement: Figure S3 — Correlation between the ratio of miR-155 and miR-425 and THRB expression in tumor vs control samples. Multiple correlation plots showing the association between the changes of microRNA and THRB levels in ccRCC samples. Lowered THRB expression in ccRCC when compared to paired control tissue is correlated with increased levels of miR-155 and miR-425. (TIF) [file pone.0097624.s003.tif]
