# Supplementary material for: Epigenetic Regulation of Thyroid Hormone Receptor Beta in Renal Cancer
Source: PLoS One. 2014 May 21;9(5):e97624. doi: 10.1371/journal.pone.0097624 (PMC4029725; doi:10.1371/journal.pone.0097624)
Supplement: Table S1 — Patient characteristics. Information on 35 ccRCC patients included in the study: histopathological diagnosis, age at disease onset and gender. (DOCX) [file pone.0097624.s004.docx]

**Supporting Table S1.** Patient characteristics.

Information on 35 ccRCC patients included in the study: histopathological diagnosis, age at disease onset and gender.

| **No.** | **Diagnosis** | **Age** | **Gender** |
| --- | --- | --- | --- |
| **1** | ccRCC, T1G1 | 46 | F |
| **2** | ccRCC, T1G1 | 63 | F |
| **3** | ccRCC, T1G1 | 73 | F |
| **4** | ccRCC, T1G1 | 77 | F |
| **5** | ccRCC, T1G1 | 73 | M |
| **6** | ccRCC, T1G1 | 43 | M |
| **7** | ccRCC, T1G2 | 72 | F |
| **8** | ccRCC, T1G2 | 77 | F |
| **9** | ccRCC, T1G2 | 62 | F |
| **10** | ccRCC, T1G1 | 63 | F |
| **11** | ccRCC, T1G2 | 78 | M |
| **12** | ccRCC, T1G2 | 78 | M |
| **13** | ccRCC, T1G2 | 52 | M |
| **14** | ccRCC, T1G2 | 68 | M |
| **15** | ccRCC, T1G2 | 77 | M |
| **16** | ccRCC, T1G2 | 35 | M |
| **17** | ccRCC, T1G2 | 55 | M |
| **18** | ccRCC, T1G3 | 52 | F |
| **19** | ccRCC, T1G3 | 52 | F |
| **20** | ccRCC, T1G3 | 58 | M |
| **21** | ccRCC, T1G3 | 77 | M |
| **22** | ccRCC, T2G1 | 46 | F |
| **23** | ccRCC, T2G1 | 52 | F |
| **24** | ccRCC, T3G3 | 65 | F |
| **25** | ccRCC, T3G3 | 65 | F |
| **26** | ccRCC, T3G3 | 53 | F |
| **27** | ccRCC, T3G3 | 71 | F |
| **28** | ccRCC, T3G3 | 70 | F |
| **29** | ccRCC, T4G1 | 70 | F |
| **30** | ccRCC, T4G1 | 64 | F |
| **31** | ccRCC, T4G1 | 54 | M |
| **32** | ccRCC, T4G1 | 66 | M |
| **33** | ccRCC, T4G2 | 52 | M |
| **34** | ccRCC, T4G3 | 84 | M |
| **35** | ccRCC, T4G3 | 84 | M |
