# Supplementary material for: Epigenetic Regulation of Thyroid Hormone Receptor Beta in Renal Cancer
Source: PLoS One. 2014 May 21;9(5):e97624. doi: 10.1371/journal.pone.0097624 (PMC4029725; doi:10.1371/journal.pone.0097624)
Supplement: Table S2 — Primers used for BSP of THRB promoter. (DOCX) [file pone.0097624.s005.docx]

**Supporting Table S2. Primers used for BSP of *THRB* promoter.**

| Forward | Reverse | Product length | Annealing temperature |
| --- | --- | --- | --- |
| mTRB-4-509U | mTRB-4-509L | 505bp | 57ºC |
| mTRB-782-1030U | mTRB-782-1030L | 248bp | 56ºC |
| mTRB-487-803U | mTRB-782-1030L | 543bp | 57ºC |
| Control-U | Control-L | 473bp | 53ºC |
