# Supplementary material for: Epigenetic Regulation of Thyroid Hormone Receptor Beta in Renal Cancer
Source: PLoS One. 2014 May 21;9(5):e97624. doi: 10.1371/journal.pone.0097624 (PMC4029725; doi:10.1371/journal.pone.0097624)
Supplement: Table S3 — Primers used in MSP-PCR and SNaPshot. (DOCX) [file pone.0097624.s006.docx]

**Supporting Table S3. Primers used in MSP-PCR and SNaPshot.**

| **Primer** | **Type of analysis** | **Sequence** |
| --- | --- | --- |
| TRB-U-Fw | MSP-PCR | TATTGGTAATTTGGTTAGAGGATTGTGT |
| TRB-U-Rev | MSP-PCR | CACACCCCTCCAAATTCTTACAACA |
| TRB-M-Fw | MSP-PCR | GGTAATTTGGTTAGAGGATCGCGC |
| TRB-M-Rev | MSP-PCR | CACCCCTCCGAATTCTTACGACG |
| mTRB2U | SNaPshot | GTTATTAATTTTGGGAGGGTA |
| SNPamp2U3 | SNaPshot | GTAGGGGGCGTTTGTAT |
| SNPamp2u3 | SNaPshot | GGGTTTGTTTGTTTTTGGT |
| SNPamp3U1 | SNaPshot | GTTTATAAAAGTGGAGAGAT |
| SNPamp3U2 | SNaPshot | GGGGATTTTGGTGTTTTAGT |
| SNPamp4U1 | SNaPshot | GTATTGGTAATTTGGTTAGAGGAT |
| SNPamp2U3 | SNaPshot | GTAGGGGGCGTTTGTAT |
| SNPamp3U2 | SNaPshot | GGGGATTTTGGTGTTTTAGT |
| SNPamp3L4 | SNaPshot | CCTCTCCGAATCCCCC |
| metTRB1L | SNaPshot | TACCCTCCCAAAATTAATAAC |
| SNPamp1L2 | SNaPshot | CTCCTAAACCACTTATAAC |
| SNPamp1L3 | SNaPshot | ATACTATAAATTTAATAACC |
| SNPamp2L3 | SNaPshot | CTCCGAACCAACGCTCC |
| SNPamp2L4 | SNaPshot | ACCAAAAACAAACAAACCC |
| metTRBL | SNaPshot | CCTACAAAATATCAACTCCTTAAAC |
| SNPamp3L2 | SNaPshot | ACTAAAACACCAAAATCCCC |
| SNPamp3L3 | SNaPshot | CAAAATCCCCGCCTCTCC |
| SNPamp3L4 | SNaPshot | CCTCTCCGAATCCCCC |
