# Supplementary material for: Epigenetic Regulation of Thyroid Hormone Receptor Beta in Renal Cancer
Source: PLoS One. 2014 May 21;9(5):e97624. doi: 10.1371/journal.pone.0097624 (PMC4029725; doi:10.1371/journal.pone.0097624)
Supplement: Table S5 — microRNA mimics and inhibitors used in the study. (DOCX) [file pone.0097624.s008.docx]

**Supporting Table S5: microRNA mimics and inhibitors used in the study.**

| assay name | assay ID | microRNA |
| --- | --- | --- |
| Ambion® Pre-miR# miRNA Precursors | PM11575 | hsa-miR-425 |
| Ambion® Anti-miR# miRNA Inhibitors | AM11575 | hsa-miR-425 |
| Ambion® Pre-miR# miRNA Precursors | PM11631 | hsa-miR-592 |
| Ambion® Pre-miR# miRNA Precursors | PM12601 | hsa-miR-155 |
| Ambion® Anti-miR# miRNA Inhibitors | AM12601 | hsa-miR-155 |
| Ambion® Pre-miR# miRNA Precursors | PM12486 | hsa-miR-185 |
| Ambion® Pre-miR# miRNA Precursors | PM11457 | hsa-miR-599 |
| Ambion® Pre-miR# miRNA Precursors | PM10337 | hsa-miR-221 |
| Ambion® Pre-miR Negative Control #1 |  |  |
